# The Electrocortical Effects of Repurposing and Reconstrual on the Regulation of Disgust

**DOI:** 10.1002/pchj.70035

**Published:** 2025-07-09

**Authors:** Chunsheng Wang, Yi Li, Tie Sun, Adjei Peter Darko, Jun Ren

**Affiliations:** ^1^ School of Psychology Zhejiang Normal University Jinhua China; ^2^ Key Laboratory of Intelligent Education Technology and Application of Zhejiang Province Zhejiang Normal University Jinhua China; ^3^ Fujian Polytechnic Normal University Fuqing China; ^4^ College of Education Zhejiang Normal University Jinhua China

**Keywords:** cognitive reappraisal, disgust, late positive potential (LPP), left negativity component (LNC), reconstrual, repurposing

## Abstract

Cognitive reappraisal serves as a pivotal strategy in emotion regulation, encompassing techniques such as repurposing and reconstrual. However, the behavioral and temporal disparities between these two reappraisal subtypes remain underexplored. This study aims to delineate these differences by comparing the psychophysiological impacts of repurposing versus reconstrual on disgust emotion regulation, employing event‐related potentials (ERPs) as the primary neurophysiological indicator. Behavioral data revealed that both strategies evoked significantly greater pleasure and less disgust compared to negative description conditions. Notably, repurposing elicited a more pronounced positive emotional shift. Electroencephalographic (EEG) findings indicated that repurposing led to a lower late positive potential (LPP) amplitude (1000–3000 ms) in frontal and parietal regions compared to reconstrual or negative descriptions. Furthermore, both strategies elicited larger left negativity component (LNC) amplitude (500–1000 ms) than negative descriptions, with repurposing demonstrating a prolonged LNC effect (1000–1500 ms) compared to reconstrual. This investigation confirms that although repurposing requires extended semantic processing resources, it exhibits superior efficacy in mitigating disgust responses. By providing direct empirical comparisons between these reappraisal modalities, the research advances our understanding of the neural mechanisms underlying cognitive emotion regulation.

## 1 Introduction

Effective emotion regulation represents the cornerstone to unlocking a life filled with mental stability and fulfillment (Gross 2002). To achieve effective emotion regulation, various strategies can be employed, including situation selection, modification, attentional deployment, cognitive reappraisal, and response modulation (Gross 2015). Among these strategies, cognitive reappraisal emerges as a more potent means of mitigating negative emotions, compared to suppression and distraction (Olatunji et al. 2020, 2017; Gross 1998). It operates by fostering the reinterpretation of emotional stimuli, thereby altering emotional responses (Buhle et al. 2014).

Numerous studies on emotion regulation have demonstrated that reappraisal represents an effective strategy of achieving emotional objectives with minimal side effects. Cognitive reappraisal has been shown to modify emotional experiences, expressions, and physiological responses, often demanding minimal cognitive effort while yielding lasting effects (Buhle et al. 2014; Morawetz et al. 2017). Moreover, the frequent practice of daily cognitive reappraisal has been associated with enhanced well‐being (McRae et al. 2012) and a reduced prevalence of mental health issues (Aldao et al. 2010).

Notably, cognitive reappraisal is not a monolithic strategy but encompasses multiple distinct substrategies. Expert taxonomies diverge significantly: for instance, Garnefski et al. (2001) identified nine strategies via the Cognitive Emotion Regulation Questionnaire, including self‐blame, acceptance, and catastrophizing. Meanwhile, Shiota and Levenson (2009) focused on two key subtypes: *detached reappraisal* (reducing emotional reactivity by focusing on nonemotional features, metaphorically termed “Turn Down the Volume”) and *positive reappraisal* (acknowledging negative events while emphasizing their positive outcomes, or “Change the Channel”) (Shiota and Levenson 2012). Other scholars have proposed additional variants, such as creative reappraisal (Wu et al. 2019), yet these frameworks often exhibit conceptual overlap or scope variation. Collectively, such classifications highlight the field's fragmentation: terminological inconsistencies and boundary ambiguities preclude the formulation of a unified “map” of reappraisal strategies (McRae 2016).

**FIGURE 1:**
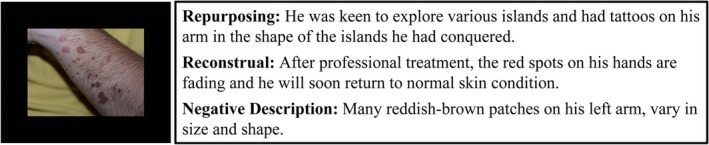
One example of disgusting pictures with three description types.

To address these theoretical challenges and clarify the underlying mechanisms, Uusberg et al. (2019) introduced the reappraisal framework, rooted in appraisal theory. Within this framework, reappraisal denotes a deliberate process of adjusting appraisal outcomes along various appraisal dimensions to modify emotional states. This process involves altering either the goal set (repurposing) or the situational construal (reconstrual) to shift emotional experience. Here, the goal set refers to active mental representations of how one desires the world to be, while the situational construal embodies of how the world actually is. The framework categorizes cognitive reappraisal into two primary strategies: reconstrual and repurposing. Reconstrual involves changing the construal of a situation to modify associated emotional responses (Uusberg et al. 2019). For example, reframing “red spots on the hands” as “fading marks that indicate improving skin condition”(see Figure 1) illustrates this strategy. Conversely, repurposing entails modifying the goal set to shift emotional responses. For instance, redefining the same red spots as “tattoos of conquered islands on a traveler's arm” (see Figure 1) exemplifies how altering motivational objectives reshapes emotional meaning. In the original scenario, the initial situational construal was “red spots on the hand,” with the goal set framing them as “red spots signify poor health and must be eliminated.” Reconstrual accomplishes cognitive reappraisal through modifying situational perceptions, whereas repurposing achieves this via goal‐set restructuring. Notably, the emphasis on structural modifications to situational construal or goal sets differentiates our target strategies from detached and positive reappraisal (Shiota 2006). The latter focus on “emotional engagement styles”—such as depersonalized observation (detached reappraisal) or silver‐lining seeking (positive reappraisal)—without undertaking structural alterations to situational interpretations or motivational frameworks. These distinctions highlight that our research examines strategies operating at the level of cognitive‐motivational restructuring.

However, these two strategies are purely theoretical constructs and lack direct empirical comparison. First, due to the lack of clear distinction in previous studies, it is challenging to determine whether findings reflect one strategy or both (Uusberg et al. 2019). Second, current research tends to prioritize the reconstrual strategy, often defining cognitive reappraisal as “altering the significance of a situation or one's emotional response (Webb et al. 2012).” This definition makes it difficult to conceptualize cognitive reappraisal solely through the lens of repurposing. Notably, the repurposing strategy is widely observed in practice, such as the adage “failure is the mother of success.” To date, no study has systematically explored the similarities and differences between repurposing and reconstrual.

The study aims to compare the emotional experience and neurophysiological responses of repurposing and reconstrual during disgust regulation. By contrasting the underlying constructs of these two strategies, we hypothesize that repurposing may afford a more comprehensive reinterpretation of emotional stimuli relative to reconstrual. Notably, a crucial marker in emotion‐related ERP research is the Late Positive Potential (LPP) (Hajcak and Foti 2020). Characterized by a sustained positive deflection in the ERP waveform, the LPP typically emerges 400 milliseconds (ms) after stimulus onset and is amplified in response to emotionally salient stimuli, such as disgust (MacNamara et al. 2022). Prior studies have shown that LPP amplitudes to disgust stimuli are significantly higher than those to neutral stimuli (Hartigan and Richards 2020; Wheaton et al. 2013). Importantly, LPP attenuation following cognitive reappraisal corresponds to the reduction in emotional reactivity during regulation (Foti and Hajcak 2008). Studies have reported lower LPP amplitudes for negative pictures during reappraisal versus passive viewing (Qi et al. 2017; Bautista et al. 2022; Foti and Hajcak 2008). Building on these findings, we propose that repurposing and reconstrual will manifest distinct LPP patterns.

In addition, we also focused on the electroencephalographic (EEG) activity when the participants understood distinct types of textual descriptions (negative descriptions, repurposing, and reconstrual) via a guided reappraisal paradigm. This paradigm directed participants to interpret disgusting pictures through textual cues (Foti and Hajcak 2008). Prior research has shown that compared to default negative descriptions, individuals employ cognitive reappraisal less frequently when processing negative pictures (Suri et al. 2015), potentially because cognitive reappraisal demands greater effort than alternative strategies (Milyavsky et al. 2019). This difficulty may stem from the need to override inherent biases—such as the automatic association between negative pictures and negative interpretations—thus making cognitive reappraisal conditions (repurposing and reconstrual) more challenging for textual comprehension than negative description conditions. The process of integrating textual and visual information further warrants attention to the N400 and Late Negativity Component (LNC), two ERP markers of semantic processing. The N400 is a negative wave that emerges between 300 and 500 ms following the onset of a critical word (Kutas and Hillyard 1984), while the LNC typically emerges after the N400 component and is generally regarded as a delayed, N400‐like component. Previous research has indicated that complex semantics elicit larger N400 and LNC amplitudes than simple semantics (Xiao et al. 2022). Furthermore, uncommon semantics elicit a larger N400 and LNC than common ones do (Kröger et al. 2013; Zhou et al. 2020). These findings suggest that the depth of semantic processing required for understanding cognitive reappraisal texts, such as repurposing and reconstrual, may be reflected in the amplitude of these ERP components.

Given the critical role of temporal dynamics in emotion regulation research, ERPs provide superior temporal resolution. This makes them ideal for exploring the temporal dynamics of reconstrual and repurposing strategies in disgust regulation (MacNamara et al. 2022). In this study, we utilized ERPs to delve into those temporal dynamics. Building on prior research, we hypothesized that repurposing would elicit lower LPP amplitudes compared to reconstrual. We further propose that understanding cognitive reappraisal texts requires deeper semantic processing than negative descriptions, which may be reflected in the N400 and LNC components.

### 1.1 Participants

Thirty‐one undergraduate students (mean age = 19.42 ± 1.31 years, 22 females) participated in the study. All were right‐handed, had normal or corrected‐to‐normal visual acuity, and reported no history of psychological or neurological disease or brain injury. A priori statistical power analysis (ANOVA: Repeated measures, within‐subjects factors, effect size *f* = 0.25, power = 0.8, α = 0.05) was conducted using G*Power 3.1.9.7 (Faul et al. 2009), indicating that a minimum sample size of 28 was required. All participants read and signed informed consent forms before the experiment and were compensated afterward. All procedures of this study adhered to the Declaration of Helsinki and were approved by the Human Research Ethics Committee of Zhejiang Normal University. All participants received 50 RMB compensation upon completion.

## 2 Methods

### 2.1 Participants

### 2.2 Materials

#### 2.2.1 Picture Materials

Ninety‐six disgusting pictures were selected from the Disgust‐Related Images database (DIRTI) (Haberkamp et al. 2017). The pictures depicted nasty bugs, infected wounds, and unhygienic scenes. To address cross‐cultural differences, 35 independent raters (mean age = 21.31 ± 2.18 years) were recruited to evaluate the valence and arousal of the pictures. The pictures were presented in a random order, and the raters were asked to rate their feelings (disgust, fear, and joy) on a scale of 1 (*none*) to 9 (*very strong*). The results revealed a significant main effect of the disgusting pictures, *F*(2, 68) = 717.62, *p* < 0.001, *η_p_* ^2^ = 0.88. Post hoc tests indicated that the most prevalent emotion evoked was disgust (*M* = 5.68, SD = 0.91), significantly higher than both fear (*M* = 3.29, SD = 0.49; *p* < 0.001, *Cohen's d* = 3.27) and joy (*M* = 2.23, SD = 0.35; *p* < 0.001, *Cohen's d* = 5.00). These findings confirm that disgusting pictures were effective in inducing disgust.

#### 2.2.2 Reappraisal Materials

For each disgusting picture, three types of sentences were prepared: repurposing, reconstrual, and negative descriptions. Following the reappraisal material generation protocol of a prior study (Foti and Hajcak 2008), 30 additional participants were recruited to generate sentences, with submissions due 5 days later. We then screened each sentence, removing unsatisfactory ones (i.e., those unrelated to disgust reduction or mismatched), merging similar sentences, refining satisfactory sentences, and standardizing them into 30–40 Chinese character statements. To ensure readability, a Chinese literature major student revised all text materials for each picture. Sentence lengths across the three reappraisal types were comparable: *M*_repurposing_ = 33.44, SD_repurposing_ = 1.38; *M*_reconstrual_ = 33.17, SD_reconstrual_ = 1.69; *M*_negative descriptions_ = 32.94, SD_negative descriptions_ = 1.25, *F*(2, 190) = 2.85, *p* = 0.06. This yielded a material library of 96 disgusting pictures, each paired with three description types. An example is provided in Figure 1.

### 2.3 Procedure

A guided reappraisal paradigm was employed, whereby participants viewed disgusting pictures paired with descriptions and provided emotional ratings (pleasure, disgust). The guided reappraisal paradigm can ensure a sufficient number of effective reappraisals within a short period. More importantly, the guiding paradigm can control the difficulty of the task and exclude the effect of cognitive load on the LPP (Foti and Hajcak 2008).

**FIGURE 2:**
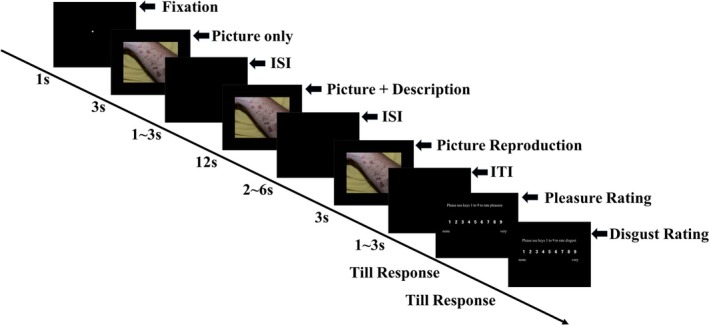
Temporal sequence of events in the study. For each trial, a white “+” fixation cross was first presented for 1 s, followed by a picture displayed for 3 s. After a blank screen lasting 1–3 s, the same picture reappeared with one of three descriptions (repurposing, reconstrual, and negative description), remaining on the screen for 12 s. This was followed by another blank screen ranging from 2 to 6 s, after which the picture alone was presented again for 3 s. Finally, after a black screen lasting 1–3 s, participants were asked to provide emotional ratings (Please use keys 1–9 to rate pleasure/disgust.) by pressing keys 1 (*none*) to 9 (*very*). ISI (interstimulus interval) and ITI (intertrial interval) denote the time intervals between stimuli and between trials, respectively.

In the study, participants first viewed a disgusting picture during the “picture only” stage. Subsequently, they were presented with one of three descriptions alongside the picture. Finally, they viewed the picture again during the “picture reproduction” stage and provided emotional ratings (pleasure and disgust) elicited by the stimulus (Figure 2).

For each participant, 96 pictures were randomly presented. Among these, every third disgusting picture was accompanied by one of three different types of descriptions. This meant that for each type of description, each participant would see 32 pictures, ensuring that each participant saw only one description for each picture.

### 2.4 EEG Recordings and Analysis

The EEG data were recorded using 64 Ag/AgCl scalp electrodes arranged according to the international 10–20 system (online bandpass filter: 0.05–100 Hz; sampling rate: 500 Hz). The data collection was facilitated by BrainVision Recorder software and BrainAmp amplifiers (BrainProducts GmbH; Munich, Germany). The maximum electrode impedance recorded was 5 kΩ. All data were based on AFz as the ground electrode and the left mastoid (TP9) as the reference electrode during recording. During data acquisition, AFz served as the ground electrode, and the left mastoid (TP9) was used as the reference electrode. Postrecording, all scalp electrode data were re‐referenced offline to the average activity of the left and right mastoids (TP9 and TP10).

The EEG data were processed offline using MATLAB 2016b (MathWorks, Natick, MA) and EEGLAB 14.1.2 (Delorme and Makeig 2004). The raw EEG data were filtered with a high‐pass cutoff of 0.1 Hz and a low‐pass cutoff of 30 Hz (Foti and Hajcak 2008; Rataj et al. 2018). For the semantic‐driven N400 and LNC analysis, the EEG data were epoched from −500 to 6000 ms relative to the onset of “picture description” (Figure 2), aligning with the temporal dynamics of semantic interpretation processes (Cao et al. 2020), with the activity from −500 to 0 ms serving as the baseline. For the emotion‐driven LPP analysis, the EEG data were epoched from −500 to 3000 ms relative to the onset of “picture reproduction” (Figure 2), again using the activity from −500 to 0 ms as the baseline (Parvaz et al. 2015; Foti and Hajcak 2008; Dennis and Hajcak 2009). This distinction in task timing (description vs. reproduction) allows dissociating semantic restructuring mechanisms (N400/LNC) from emotional modulation effects (LPP). All epoched data exhibiting large drifts were manually removed, and those contaminated by eyeblinks or eye movements were corrected using independent component analyses (ICA) (Delorme and Makeig 2004). Epoched data with errors or contamination from artifacts exceeding ±300 μV were excluded from the analysis (Foti and Hajcak 2008). Finally, the epoched data for each condition were averaged separately.

Using orthogonal selection (Luck and Gaspelin 2017), measurement parameters were determined based on ERP waveforms and topographic maps, and theoretical predictions about semantic‐cognitive versus emotional‐motivational processing. The time windows and channels of the ERP components were selected to align with their established functional signatures in prior literature, as detailed below: The amplitudes of the N400 and LNC were calculated by assessing the mean activity across nine central‐parietal electrodes: Cz, C2, C4, Pz, P2, P4, CPz, CP2, and CP4, a cluster consistently linked to semantic processing and cognitive control in prior studies (Rataj et al. 2018; Kröger et al. 2013; Zhou et al. 2018). These amplitudes were analyzed within specified time windows: the 400–500 ms window for N400 matches the classic latency of semantic integration processes (e.g., detecting word/sentence‐level anomalies), while LNC windows (500–1000 ms and 1000–1500 ms) capture sustained semantic elaboration or working memory engagement (Rataj et al. 2018; Zhou et al. 2020). These time ranges were chosen to isolate early semantic decoding (N400) from subsequent cognitive elaboration (LNC), both critical for understanding how participants reinterpret stimulus meaning.

The LPP amplitudes were quantified within distinct time windows: 400–1000 ms, 1000–2000 ms, and 2000–3000 ms (Foti and Hajcak 2008). Specifically, frontal LPP amplitudes were calculated by averaging activity across nine electrodes: F1, Fz, F2, FC1, FCz, FC2, C1, Cz, and C2 (Ma et al. 2019). Posterior‐parietal LPP amplitudes were determined by computing the mean activity of six electrodes: P1, Pz, P2, CP1, CPz, and CP2, as these regions are canonical for indexing emotional salience and motivational processing (Hartigan and Richards 2020; Wang et al. 2020).

### 2.5 Statistical Analysis

Statistical analysis was conducted using SPSS 27.0 (IBM, Armonk, NY, USA). A three‐way repeated‐measures ANOVA (with factors of repurposing, reconstrual, and negative description) was performed to analyze emotional ratings (including pleasure and disgust) and ERP components (N400, LNC, and LPP). For violations of sphericity, the Greenhouse–Geisser correction was applied to adjust the degrees of freedom for F‐statistics. Following significant main effects or interactions, post hoc pairwise comparisons used the Bonferroni–Holm method for *p* value adjustment. Effect sizes are presented as partial eta‐squared (*η_p_* ^2^) and *Cohen's d*.

## 3 Results

### 3.1 Behavioral Results

**FIGURE 3:**
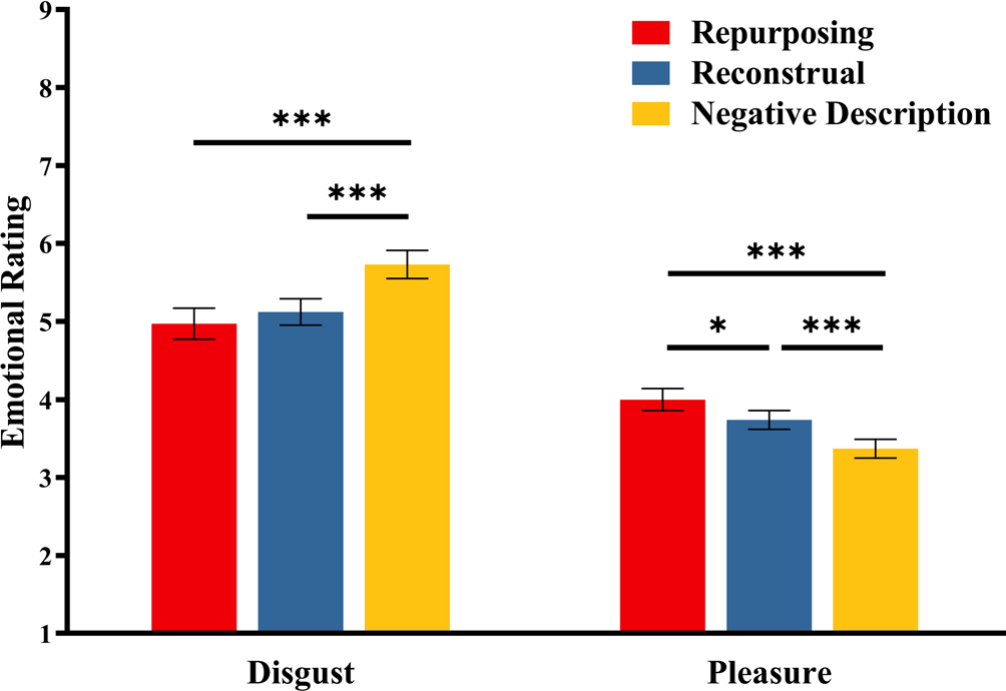
Emotional ratings (disgust, pleasure) for three description types. Error bars indicate the standard deviation. *p < 0.05, ***p < 0.001.

The emotional rating results (disgust and pleasure) are presented in Figure 3. A single‐factor repeated‐measures ANOVA was conducted to analyze ratings across three description types.

For disgust ratings, a significant main effect was observed, with *F*(2, 60) = 26.34, *p <* 0.001, *η_p_* ^2^ = 0.47. Post hoc comparisons (Bonferroni–Holm corrected) revealed significantly lower disgust ratings for repurposing (*M* = 4.97, SD = 0.20, *p <* 0.001, *Cohen's d* = −1.14) and reconstrual (*M* = 5.12, SD = 0.17, *p <* 0.001, *Cohen's d* = −0.96) compared to negative descriptions (*M* = 5.73, SD = 0.18). No significant difference was found between repurposing and reconstrual conditions (*p =* 0.42, *Cohen's d* = −0.27).

For pleasure ratings, a significant main effect was found, with *F*(2, 60) = 24.41, *p <* 0.001, *η_p_* ^2^ = 0.45. Post hoc comparisons (Bonferroni–Holm corrected) showed significantly higher pleasure ratings for repurposing (*M* = 4.00, SD = 0.14, *p <* 0.001, *Cohen's d* = 1.13) and reconstrual (*M* = 3.74, SD = 0.12, *p <* 0.001, *Cohen's d* = 0.91) versus negative descriptions (*M* = 3.37, SD = 0.12). Further, pleasure ratings for repurposing were significantly higher than those for reconstrual (*p =* 0.034, *Cohen's d* = 0.49).

### 3.2 ERP Results

#### 3.2.1 N400 And LNC

**FIGURE 4:**
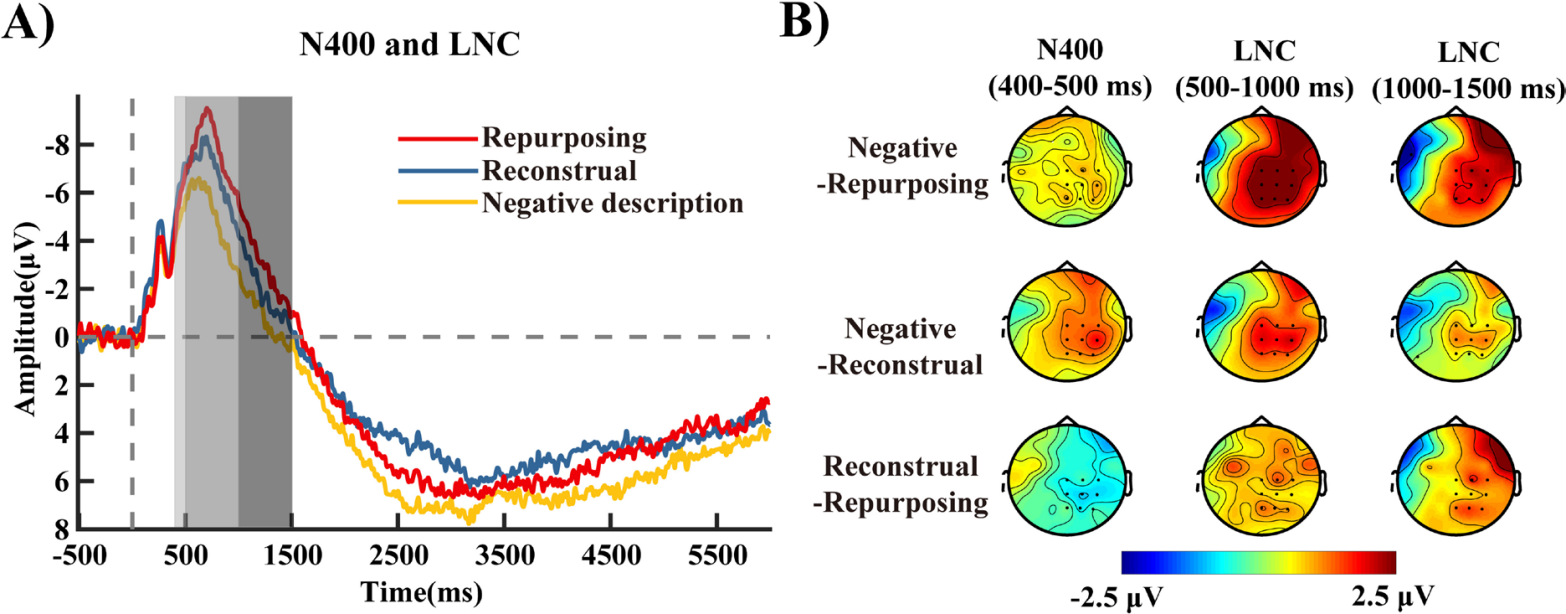
ERP waveforms and topographical maps of N400 and LNC (*n* = 31). (A) Grand‐averaged event‐related potential (ERP) waveforms were time‐locked to the onset of “picture description” over nine electrodes (Cz, C2, C4, Pz, P2, P4, CPz, CP2, and CP4). (B) Scalp topography of the difference between negative description and repurposing (top), the difference between negative description and reconstrual (middle), as well as the difference between reconstrual and repurposing (bottom) in the 400–500 ms (left), 500–1000 ms (middle), and 1000–1500 ms (right) windows.

For N400 (400–500 ms), a significant main effect was found, *F*(2, 60) = 3.32, *p =* 0.043, *η_p_* ^2^ = 0.1 (Figure 4). Post hoc tests revealed that the reconstrual condition (*M* = −6.50, SD = 1.11) elicited significantly larger N400 amplitudes than the negative description condition (*M* = −5.10, SD = 1.07, *p =* 0.025, *Cohen's d* = −0.51). No significant differences were found between the repurposing condition (*M* = −5.94, SD = 0.98) and either the negative description (*p =* 0.47, *Cohen's d* = −0.26) or reconstrual (*p =* 0.42, *Cohen's d* = 0.27) conditions.

For LNC (500–1000 ms), a significant main effect was found, *F*(2, 60) = 9.81, *p <* 0.001, *η_p_* ^2^ = 0.25 (Figure 4). Post hoc comparisons (Bonferroni–Holm corrected) showed that both the repurposing (*M* = −6.88, SD = 0.99, *p =* 0.002, *Cohen's d* = −0.70) and reconstrual (*M* = −5.96, SD = 1.09, *p =* 0.018, *Cohen's d* = −0.53) conditions elicited significantly larger LNC amplitudes than the negative description condition (*M* = −4.29, SD = 1.04). No significant difference was found between repurposing and reconstrual conditions (*p =* 0.42, *Cohen's d* = −0.30).

For LNC (1000–1500 ms), a significant main effect was found, *F*(2, 60) = 3.94, *p =* 0.025, *η_p_* ^2^ = 0.12 (Figure 4). Post hoc comparisons (Bonferroni–Holm corrected) revealed that the repurposing condition (*M* = −2.81, SD = 0.89, *p =* 0.045, *Cohen's d* = −0.46) significantly elicited larger LNC amplitudes than the negative description condition (*M* = −0.94, SD = 0.91). No significant differences were found between the reconstrual condition (*M* = −1.99, SD = 0.83) and either the negative description (*p =* 0.47, *Cohen's d* = −0.26) or repurposing (*p =* 0.42, *Cohen's d* = 0.27) conditions.

#### 3.2.2 Frontal LPP

**FIGURE 5:**
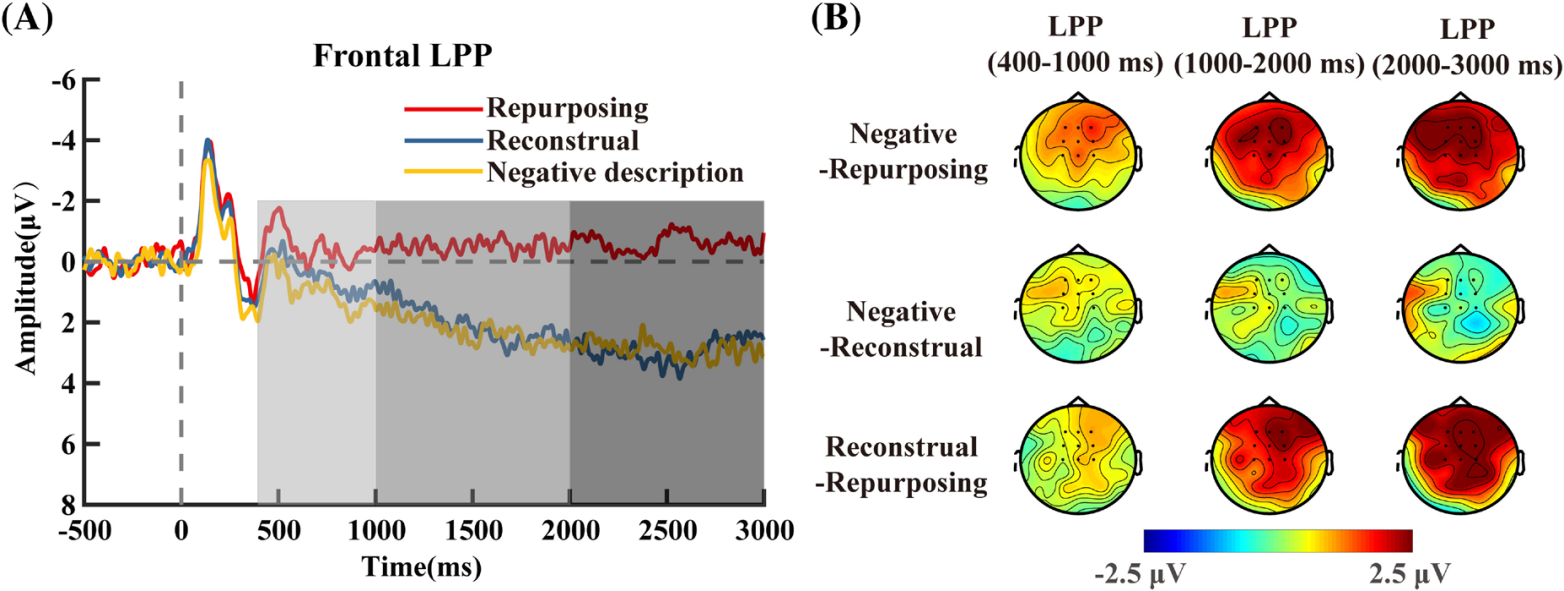
ERP waveforms and topographical maps of frontal LPP (*n* = 31). (A) Grand‐averaged event‐related potential (ERP) waveforms were time‐locked to the onset of “picture reproduction” over nine electrodes (F1, Fz, F2, FC1, FCz, FC2, C1, Cz, and C2). (B) Scalp topography of the difference between negative description and repurposing (top), the difference between negative description and reconstrual (middle), as well as the difference between reconstrual and repurposing(bottom) in the 400–1000 ms (left), 1000–2000 ms (middle), and 2000–3000 ms (right) windows.

For the frontal LPP (400–1000 ms), a significant main effect was observed, with *F*(2, 60) = 3.50, *p =* 0.037, *η_p_* ^2^ = 0.10 (Figure 5). Post hoc comparisons (Bonferroni–Holm corrected) revealed that repurposing (*M* = −0.42, SD = 0.80) elicited significantly lower LPP amplitudes compared to the negative description condition (*M* = 1.04, SD = 0.93, *p* = 0.036, *Cohen's d* = −0.48). No significant differences were found between the reconstrual condition (*M* = 0.60, SD = 0.93) and either the negative description (*p* = 1.00, *Cohen's d* = −0.16) or repurposing (*p =* 0.38, *Cohen's d* = 0.28) conditions.

For the frontal LPP (1000–2000 ms), a significant main effect emerged, *F*(2, 60) = 8.29, *p* < 0.001, *η_p_* ^2^ = 0.22 (Figure 5). Post hoc comparisons (Bonferroni–Holm corrected) showed that the repurposing condition (*M* = −0.46, SD = 1.03) elicited significantly lower LPP amplitudes than both the reconstrual (*M* = 2.20, SD = 0.93, *p =* 0.02, *Cohen's d* = −0.53) and negative description (*M* = 2.35, SD = 0.89, *p* = 0.003, *Cohen's d* = −0.66) conditions. No significant difference was found between reconstrual and negative description conditions (*p* = 1.00, *Cohen's d* = −0.04).

For the frontal LPP (2000–3000 ms), a significant main effect was found, *F*(2, 60) = 9.58, *p <* 0.001, *η_p_* ^2^ = 0.24 (Figure 5). Post hoc comparisons (Bonferroni–Holm corrected) showed that the repurposing condition (*M* = −0.63, SD = 0.94) elicited significantly lower LPP amplitudes than both the reconstrual (*M* = 3.12, SD = 0.81, *p =* 0.002, *Cohen's d* = −0.62) and negative description (*M* = 2.96, SD = 0.98, *p =* 0.005, *Cohen's d* = −0.69) conditions. No significant difference was found between reconstrual and negative description conditions (*p* = 1.00, *Cohen's d* = 0.03).

#### 3.2.3 Posterior‐Parietal LPP

**FIGURE 6:**
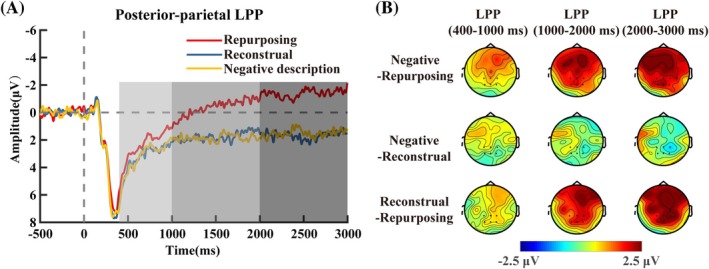
ERP waveforms and topographical maps of posterior‐parietal LPP (*n* = 31). (A) Grand‐averaged event‐related potential (ERP) waveforms were time‐locked to the onset of “picture reproduction” over six electrodes (P1, Pz, P2, CP1, CPz, and CP2). (B) Scalp topography of the difference between negative description and repurposing (top), the difference between negative description and reconstrual (middle), as well as the difference between reconstrual and repurposing (bottom) in the 400–1000 ms (left), 1000–2000 ms (middle), and 2000–3000 ms (right) windows.

For the posterior‐parietal LPP (400–1000 ms), there was not a significant main effect, *F*(2, 60) = 1.60, *p =* 0.21, *η_p_* ^2^ = 0.05 (Figure 6).

For the posterior‐parietal LPP (1000–2000 ms), a significant main effect was found, *F*(2, 60) = 4.70, *p =* 0.019, *η_p_* ^2^ = 0.14 (Figure 6). Post hoc comparisons (Bonferroni–Holm corrected) showed that the repurposing condition (*M* = −0.36, SD = 0.97) elicited significantly lower LPP amplitudes than the negative description condition (*M* = 1.90, SD = 0.82, *p =* 0.043, *Cohen's d* = −0.47). No significant differences were found between the reconstrual condition (*M* = 1.88, SD = 0.84) and either the negative description (*p* = 1.00, *Cohen's d* = −0.04) or repurposing (*p =* 0.09, *Cohen's d* = 0.53) conditions.

For the posterior‐parietal LPP (2000–3000 ms), a significant main effect was found, *F*(2, 60) = 5.46, *p =* 0.007, η*_p_* ^2^ = 0.15 (Figure 6). Post hoc comparisons (Bonferroni–Holm corrected) showed that the repurposing condition (*M* = −1.46, SD = 0.92) elicited significantly lower LPP amplitudes than both the reconstrual (*M* = 1.77, SD = 0.84, *p =* 0.044, *Cohen's d* = −0.47) and negative description (*M* = 1.54, SD = 0.93, *p =* 0.026, *Cohen's d* = −0.51) conditions. No significant difference was found between the reconstrual and negative description conditions (*p* = 1.00, *Cohen's d* = 0.04).

## 4 Discussion

The present study employed event‐related potentials (ERPs) to elucidate the temporal dynamics of repurposing and reconstrual in regulating disgust. Concerning emotional ratings, both repurposing and reconstrual outperformed negative descriptions, effectively boosting pleasure ratings and reducing disgust ratings. Notably, repurposing was more effective than reconstrual in enhancing pleasure. Furthermore, repurposing reduced the frontal and posterior‐parietal LPP, while reconstrual did not. Specifically, participants showed larger negative components (N400, LNC) when processing repurposing and reconstrual compared to negative descriptions, with temporal differences. The findings indicated that repurposing and reconstrual exhibited distinct effects on emotion regulation, as evidenced by changes in subjective experiences and electrophysiological responses.

Consistent with previous research findings, cognitive reappraisal is effective in regulating disgust (Gross 1998; Olatunji et al. 2017; Schubert et al. 2020), particularly when employing strategies such as repurposing and reconstrual. Furthermore, we observed varying impacts of these strategies on adjusting ratings of pleasure and disgust. More specifically, repurposing was more effective than reconstrual in enhancing pleasure ratings, whereas it was equally effective as reconstrual in reducing disgust ratings.

Consistent with previous studies (Foti and Hajcak 2008), ERP results showed that changes in narrative content are sufficient to induce modulations in the LPP. Notably, the LPP activity (1000–2000 ms and 2000–3000 ms) was lower for the repurposing condition than for both the reconstrual and negative description conditions. It is generally believed that the decline in LPP is related to a decrease in emotional response caused by emotion regulation (MacNamara et al. 2022). The amplitude of the LPP conveys the significance of the stimulus and the subsequent stimulation of the motivational system (Hajcak and Foti 2020). Thus, the LPP results suggest that repurposing has a better effect on deconstructing stimuli and establishing a more positive meaning than reconstrual.

Beyond LPP differences, variations in N400 and LNC were also observed among participants comprehending three types of textual descriptions. Notably, participants exhibited larger amplitudes of the N400 and LNC during tasks involving repurposing and reconstrual, compared to the condition when processing negative descriptions. Consistent with previous research, complex semantics evoke significantly larger amplitudes of the N400 and LNC than simple semantics (Xiao et al. 2022), and uncommon semantics elicit greater amplitudes than common semantics (Kröger et al. 2013; Zhou et al. 2020; Rataj et al. 2018). Furthermore, studies have found that cognitive reappraisal is more difficult (Milyavsky et al. 2019) and less common (Suri et al. 2015) than negative descriptions. In addition, temporal differences were observed in the amplitude changes, with repurposing predominantly affecting later time windows (500–1000 ms and 1000–1500 ms) and reconstrual exhibiting a more pronounced effect in earlier time windows (400–500 ms and 500–1000 ms). Thus, conceptually, the differences between these two strategies may underlie their distinct effects: reconstrual aligns with the default negative picture context, whereas repurposing deviates from this to construct a distinct positive scenario. Studies have shown that the increase in duration of the LNC effect is related to higher cognitive demands (DiStefano et al. 2019; Li et al. 2014). Therefore, the prolonged LNC effect for repurposing compared to reconstrual may be attributed to the higher cognitive demands associated with detaching from the original context during repurposing, which necessitates more extended semantic processing.

This study had some limitations that offer directions for future research. First, this study primarily focused on core disgust, and it remains unclear whether the results can be generalized to moral disgust. Evidence suggests that core and moral disgusting pictures evoke distinct neural activity patterns throughout the stages of information processing (Zhang et al. 2015). Consequently, the effects of repurposing and reconstrual on regulating moral disgust may occur through different temporal processes. Second, we used a paradigm to naturally guide participants to apply various reappraisal strategies to alleviate disgust emotions. However, it is unclear how to naturally guide individuals to adopt repurposing or reconstrual strategies to reduce disgust feelings. We hypothesize that spontaneous cognitive reappraisal may be associated with the formation of new associations (Ren et al. 2020). More importantly, there are numerous scenarios involving proximity to disgust‐inducing situations (e.g., doctors treating patients, garbage disposal, anatomy training) or excessive disgust symptoms in certain psychological disorders where reducing disgust is necessary. For instance, disgust is a prominent symptom of eating avoidance disorder, and emphasis on proximity motivation or repeated exposure has limited effectiveness in reducing disgust (Harris et al. 2019). Repurposing may effectively reduce aversive symptoms by reshaping people's perceptions of food, thereby altering their food preferences.

In this study, we discovered that repurposing demands more semantic processing time but is more effective than reconstrual in regulating disgust emotions. Our findings provide direct empirical comparisons between reconstrual and repurposing strategies and may offer valuable insights into the underlying mechanisms of cognitive reappraisal. Furthermore, our research establishes a theoretical foundation for the practical application of repurposing or reconstrual strategies to enhance or even resolve disgust‐related dilemmas.

## Conflicts of Interest

The authors declare no conflicts of interest.
